# Notes on Current Literature

**Published:** 1881-10

**Authors:** 


### BOOK REVIEWS, NOTICES, ETC.

In compliance with the expressed wish of some of our readers, we shall give, from time to time, a short list of the most prominent and interesting books published, pertaining to medicine; also note a few of the more interesting essays published in the journals. We can give no more space than suffices to mention subject and author; hence, will attempt no *review* of their subject-matter. This is done to keep our readers acquainted, without trouble to themselves, with the current medical literature, and so facilitate their purchasing such works as they may desire.

We would respectfully request all medical publishing houses to keep us informed, from time to time, as to the new books they have, either " in press or just out."

### NOTES ON CURRENT LITERATURE.

*Recently Published by Wm. Wood & Co.*

LECTURES ON DIAGNOSIS AND TREATMENT OF DISEASES OF THE CHEST, THROAT AND NASAL CAVATIES. By *E. Fletcher Ingals.* pp. 437; price $4.00.

LECTURES ON SURGICAL DISORDERS OF THE URINARY ORGANS. 2d Edition. By *Reginald Harrison.* pp. 400; price $5.00.

DISEASES OF THE BLADDER. *Coulson.* (July Volume of Library Series.)

INDIGESTION, BILIOUSNESS, GOUT, ETC. *Fothergill.* pp. 316; price $2.25.

THE SYMPATHETIC DISEASES OF THE EYE. By *Ludwig Mauthner.* Translated from the German. *In Press.*

*Recently Published by H. C. Lea's Son & Co.*

A PRACTICAL TREATISE ON IMPOTENCE, STERILITY, ETC. By *S. W. Gross.* pp. 174; price $1.50.

A PRACTICAL TREATISE ON ELECTRICITY IN ITS APPLICATION TO MEDICINE. By *Roberts Bartholow.* pp. 270; price $2.50.

LECTURES ON DISEASES OF THE NERVOUS SYSTEM, ESPECIALLY IN WOMEN. By *S. Weir Mitchell.* pp. 250; price $1.75.

*D. Appleton & Co.*

THE APPLIED ANATOMY OF THE NERVOUS SYSTEM. *A. L. Ramy.* pp. 500; price $4.00.

*Priesley Blakiston.*

A TEXT-BOOK OF MODERN MIDWIFERY. By *Rodney Glisan.* pp. 640; price $4.00.

*Lindsay & Blakiston.*

PRACTICE OF MEDICINE AND SURGERY APPLIED TO DISEASES AND ACCIDENTS INCIDENT TO WOMEN. By *W. H. Byford.* pp. 682; price $5.00.

*Boericke & Tafel. In Press.*

RAUE ON PATHOLOGY AND THERAPEUTICS. 2d Edition.

THE HUMAN EAR AND ITS DISEASES. By *Wm. H. Winslow.*

NORTON'S (formerly Allen & Norton) OPHTHALMIC THERAPEUTICS. 2d Ed.

WORCESTER ON INSANITY.

NOTES ON THERAPEUTICS. *G. Pecholier*, writes (*Gaz Med. de Paris*) on Physiological Action of Hellebore. *E. T. Riechert*, (*N. Y. Med. J.*) on Amyl nitr. as Powerful Cardiac Stimulant. *J. Ossikovszky*, points out Similarity between Symptoms of Phosphorus and Chronic Hepatitis.

TOXICOLOGY. *A. J. Alliott*, (*Brit. M. J.*) Case of Poisoning by Arum Maculatum. *J. A. Bull*, (*Boston M. & S. J.*) Strychnia Poison treated with Cannabis Indica and Chl. Hydrate. *N. Grattan*, (*London Lancet*) Belladonna Poison treated with Philocarpin. *J. Hudson*, (*Brit. M. J.*) Belladonna Poisoning treated with Physostigma. *A. D. MacDonald*, (*Edinb. M. J.*) a Case of Hydro-chloric Acid Poisoning, and Some Points in Practical Therapeutics. *E. R. Mansell*, (*Lancet*) Chloral *vs.* Strychnia. *J. M. DaCosta*, (*Phila. Med. Times*) writes on Arsenical Paralysis. *Hildebrandt*, (*Deutsche Med. Wchnshr.*, Berlin, 1881) writes on Poisoning by Carbolic Acid.

OPIUM. *J. L. Beerdeen*, (*Mich. M. News*) writes on Jaborandi and Belladonna in Opium Poisoning. *J. W. Bull*, (*Boston M. & S. J.*) Opium Poisoning treated with Atropia. *Ford*, (*St. Louis M. and S. J.*) on Atropia Antagonism to Opium. *H. H. Kahe*, (*Med. and S. Rep., Phila.*) on Rapid and Easy Cure of a Case of Morphia Habit of Twelve Years' Standing; amount used per day, sixteen grains. *C. M. Nutt*, (*Louisville M. News*) Alarming Narcosis from a small dose of Morphia. *T. Wayne*, (*Indian Med. Gaz., Calcutta*) Treatment of Opium Eaters.

SCARLATINA. Cold Baths, etc., are recommended in a work by *L. Duchesne.*

BRIGHT'S DISEASE. *T. S. Dabney* (*Indep. Pract., N. Y.*) recommends Apocynum Can. *J. E. Atchinson*, inquires (*Am. Jr. Med. Scs.*) if Potas. Iod. may not excite Bright's Disease.

DIPHTHERIA. *A. M. Williamson* (*Cinn. Lancet and Clinic*) recommends Persulph. of Iron. *Boine, Boon* and others, recommend Tannic Acid. *E. Bonchut* (*Compt. rend. Acad. de Paris*) Local Application of Papine. (*Med. Rec., N. Y.*) *Whaler* has tried Jaborandi. *Forest* and *K. Dehio* believes in Philacorpine; others in its muriate.

SEPTICÆMIA, ETC. *T. J. Burrill*, writes (*Am. Nat* 1381, V. XV.) on Bacteria as a Cause of Disease in Plants. *A. Clauveau*, "Ferments and Virus." *L. A. Stimson*, (*Med. Record*) Acute Septicæmia; Influence of Germs in the Putrefactive Process.

PASTEUR'S ADDRESS, before the recent International Congress, on Germs, etc., is reprinted in *N. Y. Medical Record* of August 27th, 1881.

